# Complex Portal 2018: extended content and enhanced visualization tools for macromolecular complexes

**DOI:** 10.1093/nar/gky1001

**Published:** 2018-10-24

**Authors:** Birgit H M Meldal, Hema Bye-A-Jee, Lukáš Gajdoš, Zuzana Hammerová, Aneta Horáčková, Filip Melicher, Livia Perfetto, Daniel Pokorný, Milagros Rodriguez Lopez, Alžběta Türková, Edith D Wong, Zengyan Xie, Elisabeth Barrera Casanova, Noemi del-Toro, Maximilian Koch, Pablo Porras, Henning Hermjakob, Sandra Orchard

**Affiliations:** 1 European Bioinformatics Institute (EMBL-EBI), European Molecular Biology Laboratory, Wellcome Genome Campus, Hinxton, Cambridgeshire CB10 1SD, UK; 2 Department of Biochemistry and National Centre for Biomolecular Research, Faculty of Science, Masaryk University, Kamenice 5, 625 00 Brno-Bohunice, Czech Republic; 3 Department of Genetics, Stanford University School of Medicine, Stanford, CA 94305-5477, USA; 4 Chongqing Key Laboratory on Big Data for Bio Intelligence, Chongqing University of Posts and Telecommunications, No. 2 Chongwen Road, Nan’an District, Chongqing 400065, China; 5 Novartis Institutes for BioMedical Research (NIBR), Basel, Canton of Basel-Stadt, Switzerland; 6 State Key Laboratory of Proteomics, Beijing Proteome Research Center, National Center for Protein Sciences (Beijing), Beijing Institute of Life Omics, Beijing 102206, China

## Abstract

The Complex Portal (www.ebi.ac.uk/complexportal) is a manually curated, encyclopaedic database that collates and summarizes information on stable, macromolecular complexes of known function. It captures complex composition, topology and function and links out to a large range of domain-specific resources that hold more detailed data, such as PDB or Reactome. We have made several significant improvements since our last update, including improving compliance to the FAIR data principles by providing complex-specific, stable identifiers that include versioning. Protein complexes are now available from 20 species for download in standards-compliant formats such as PSI-XML, MI-JSON and ComplexTAB or can be accessed via an improved REST API. A component-based JS front-end framework has been implemented to drive a new website and this has allowed the use of APIs from linked services to import and visualize information such as the 3D structure of protein complexes, its role in reactions and pathways and the co-expression of complex components in the tissues of multi-cellular organisms. A first draft of the complete complexome of *Saccharomyces cerevisiae* is now available to browse and download.

## INTRODUCTION

All biological processes rely on the interaction of proteins with other cellular components and in many cases these proteins function as part of macromolecular complexes. These complexes may be composed only of proteins or also contain nucleic acids and/or small molecules. Complexes can be stable or transient in nature and vary in their function and composition depending on the tissue type, developmental stage and biological process. Many complexes have been well characterized by experimental methods while the existence or composition of other complexes has been inferred from related evidences.

The Complex Portal (www.ebi.ac.uk/complexportal) is a manually curated, encyclopaedic resource that summarizes all functional aspects of stable macromolecular complexes (1). Since our last update, we have improved the data structure by providing more data download formats, new identifiers and expanded our data content in terms of both, the number of complexes curated and the species represented in the resource. We have significantly improved interoperability with other resources, such as the Gene Ontology (2), and updated the website to provide better data access and more visualization options, all making the Complex Portal more fully compliant with the FAIR principles of Findability, Accessibility, Interoperability and Reusability (3).

The characterization of the complex composition in a model organism can be used to predict the existence of similar complexes in other species (4). Such inferences can contribute to our understanding of evolutionary development at the molecular level. We are currently developing a tool that can predict orthologous complexes in other species as well as find homologous complexes that have already been curated in the Complex Portal.

## CONTENT

### Curation update

**Figure 1.**
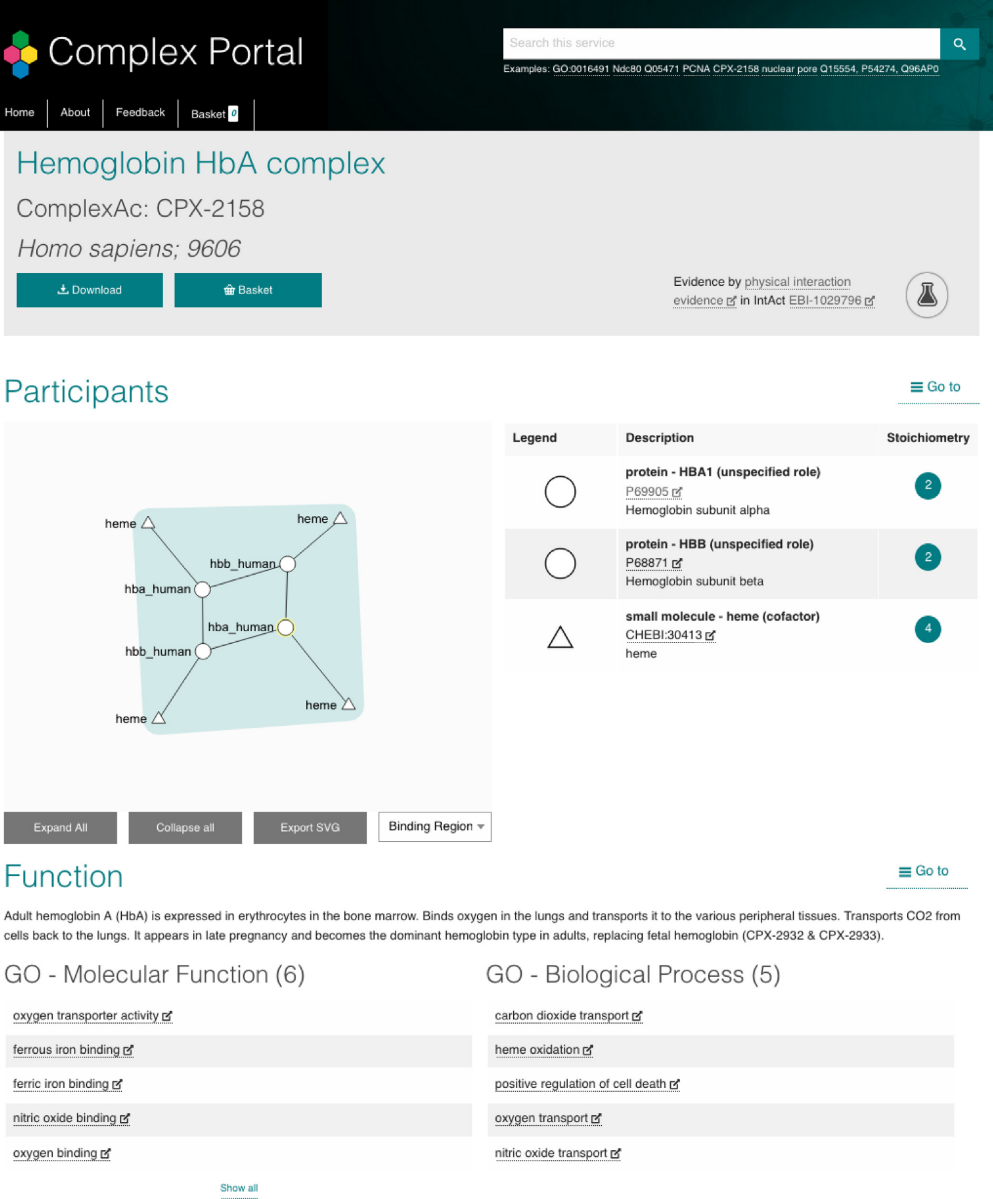
Screenshot of the details page of Haemoglobin HbA complex (CPX-2158).

The working definition of a protein complex is that it should contain two or more macromolecules for which there is evidence (experimental or inferred) that these molecules stably interact with each other and have a demonstrated molecular function. Each entry (Figure 1) describes the composition, topology and function of a single complex from a defined species and links out to external databases that provide domain-specific information of the complex, such as structural details from PDB (5) or the role of the complex in metabolic reactions or signalling pathways in Reactome (6). A complex is uniquely defined by its protein components using canonical UniProt identifiers, i.e. the protein sequence selected as the reference sequence in a UniProtKB/Swiss-Prot entry. Isoform-specific complex variants are only created when the function of the complex is specific for a given isoform. The Cyclin L-CDK11 complexes provide interesting examples where this is the case (CPX-341, CPX-343, CPX-344, CPX-347). Complex participants are described using the UniProtKB identifiers for post-processed chains when complexes are formed only by a specific cleavage product, for example the amyloid-beta protein 40/42 heterodimer (CPX-1062). Post-transcriptional modifications are described only if they are essential to the formation of the complex as, for example, in the case of the formation of the SMAD1 homotrimer (CPX-144).

The Complex Portal currently focuses on curating stable protein complexes from a limited number of model organisms to provide a reference from which complexes in other species can be inferred and the evolutionary development of a conserved functional unit can be traced. As of release 219 (8 October 2018), 2472 complexes have been curated and released.

Recent curation priorities have included:

- Mammalian complexes with available 3D structures. This is a long-term program in collaboration with CEITEC and PDBe. 297 curated mammalian complexes have crystal structures, 248 of them are human.
- Human and mouse epigenetic complexes. This set can be accessed by searching on the high-level Gene Ontology term ‘chromatin organization‘ (GO:0006325)
- *Saccharomyces cerevisiae*: All known complexes (589) for the budding yeast have been curated, in collaboration with UniProt (7) and the Saccharomyces Genome Database (8). Newly described yeast complexes are being added into SGD and the Complex Portal in direct collaboration.
- *Arabidopsis thaliana*: 221 hormone signalling complexes of *Arabidopsis thaliana* are the first plant complexes to be added to the database.
- *Escherichia coli*: known *E. coli* complexes are being curated with a view to completion of the complexsome in 2019.

### Quality control

All entries in the Complex Portal are manually annotated and carefully checked by a second curator before publication. Entries are standards compliant, for example using stable identifiers (UniProt for proteins, ChEBI for small molecules (9), RNAcentral for ncRNAs (10) and controlled vocabularies such as the Gene Ontology and PSI-MI ontology (11). Curators work to a detailed curation manual that is constantly updated and reviewed as we discover new data concepts. A summary of our curation practices is available via the <Documentation> tile on the public website; this enables users to understand the annotation process. We have implemented new syntax checks that are run before each release cycle, making the data more robust to human error. Following strict rules and quality control checks means that our entries are consistent and of high quality.

### Complex identifiers and versions

The new complex-specific stable identifiers have a version number that can be incrementally increased to indicate a major change to the underlying record, thus making the resource more fully compliant with the FAIR standard. Identifiers are of the type: CPX-xxxx.y, where xxxx is the running number and *y* the version. The previously published EBI-xxxxxxx identifiers are retained as secondary identifiers and can still be retrieved as they remain in the download files and are indexed in the webservice. The new identifiers also provide an easy distinction from IntAct experimental evidences with which they previously shared an identifier space. A complex version will be incrementally increased if the composition of the complex has changed, for example additional participants have been added. A change in stoichiometry will currently not trigger reversioning.

If a complex has been obsoleted without replacement, the accession number (AC) will no longer be searchable in the current release files or the website but will remain accessible from the archive on our ftp site (ftp://ftp.ebi.ac.uk/pub/databases/intact/complex/current). If a complex is split into several new entries the original AC will be retained as a secondary AC on the new complexes and, similarly, if two complexes are merged, the original AC from one complex becomes a secondary AC on the retained and updated complex. The website only displays the current AC whilst previous entry versions and obsoleted ACs are stored in the archive that can be retrieved from the ftp site.

### Evidence and data provenance

Many of the curated complexes are unambiguous in content and 63% are supported by good experimental evidence, which is collated and cross-referenced to the IntAct molecular interaction database, PDBe or EMBD (23). In many cases, alternative compositions exist for a complex, often caused by proteins that arose from gene duplications. Each possible variant is curated as long as there is reason to believe these exist in nature. Other complexes, however, have little or no experimental evidence and their component content is currently poorly understood. Curator judgement is often required to make a decision on the essential core components of a complex, and it is accepted that many of these entities will have to be revisited and updated as new information on their composition becomes available, with a new version of a complex being issued when appropriate. All entries are annotated with Evidence and Conclusion Ontology (ECO) codes (12) that identify the level of experimental evidence that exists for any given complex. New codes that are specific for complexes and pathways have been added to the ontology:

- *ECO:0000353 physical interaction evidence used in manual assertion* has been retained to indicate cases where the complex is fully experimentally validated.
- *ECO:0005610 biological system reconstruction evidence based on homology evidence used in manual assertion* and appropriate child terms are used to indicate orthologous complexes (inter-species comparison) and paralogous complexes (intra-species comparison where the variants are related to proteins that arose from gene duplications but retain the same function) if experimental evidence exists for a related complex not only containing orthologous/paralogous participants but that also retains compositional and functional homology.
- *ECO:0005543 biological system reconstruction evidence by experimental evidence from mixed species used in manual assertion* is used if the researcher has used constructs from several species to reconstruct a complex and if the orthologous sequences are identical or very similar.
- *ECO:0005547 biological system reconstruction evidence based on inference from background scientific knowledge used in manual assertion* describes complexes where the expert community believes the complex exists but only limited or circumstantial experimental evidence exists (e.g. pharmacological or ligand-binding assays).

### Variants, molecule sets and complexes as participants

Complex variants can be the result of (a) duplication of one or more genes, (b) the existence of isoforms that result in different functions of the complex or (c) compositional variations related to tissue type or developmental stage of the organism. In these cases, we normally create separate entries, keeping the root recommended name constant and adding an extension that describes the variation (either using terms used by the community or the gene symbols that vary). In a very limited number of cases the possible number of combinations is very large, leading to a combinatorial explosion in the number of possible variants, e.g. the yeast ribosomal sub-complexes (CPX-1599 & CPX-1601) in which most of the subunits have 2 paralogous proteins. In these cases, the protein paralogues are grouped as molecule sets. Each set only contains proteins that have the same evolutionary origin and the same function. The resource now also includes examples of hierarchical builds of complexes assembled from functionally active sub-complexes, for example the yeast CMG-Pol epsilon complex (CPX-1556).

## WEBSITE SEARCH AND DATA VISUALIZATION

The original Complex Portal website (1) consisted of a simple, mainly text-based presentation and data access was provided only by ftp. This has now been updated to provide a dynamic, interactive, responsive user interface, created using a component-based JS front-end framework served by an enhanced REST API (https://www.ebi.ac.uk/intact/complex-ws/). The layout and functionality of the site has been developed using iterative user-centric design principles. 2D visualization of the topology of each complex is supplied by the ComplexViewer (13) which provides a scalable vector graphic (SVG) element within a web page. This viewer enables the visualization of the topology of complexes containing multiple participants, sequence features such as binding domains relevant to complex formation, a range of biomolecules as interactor types (proteins, small molecules, nucleic acids, sub-complexes), discontinuous sequence features and stoichiometry information.

The existing simple search option has been supplemented by an organism-centric view which enables researchers to download all the complexes from a particular species in a choice of formats. This can be accessed from one of a series of ‘tiles’ visible on the front-page, designed as easy-to-use entry points. Other tiles lead users to training materials, documentation, a ‘basket’ (a user-defined list of stored complexes that can be populated from the individual complex details pages), and a webform for curation requests or general feedback about the service.

**Figure 2.**
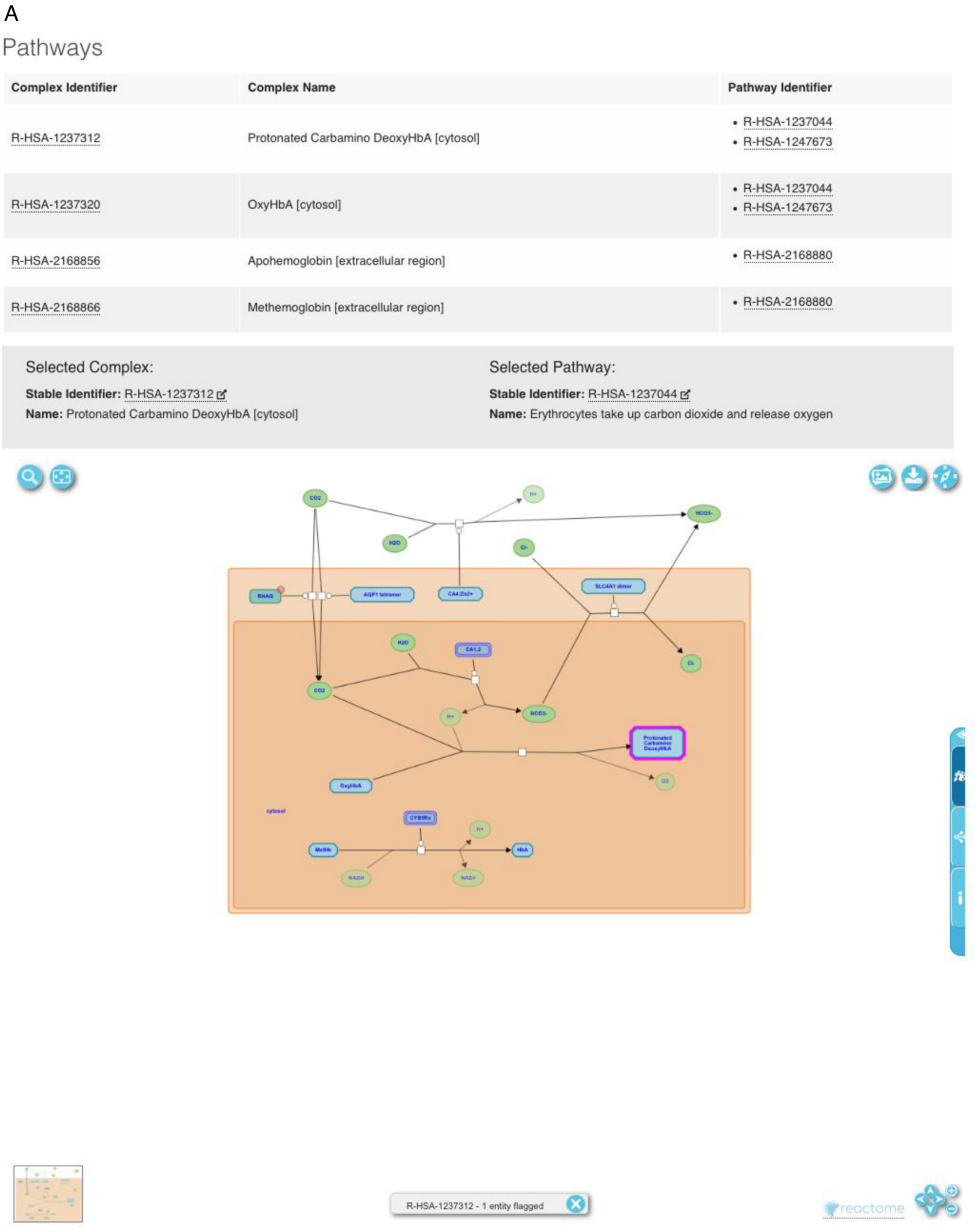

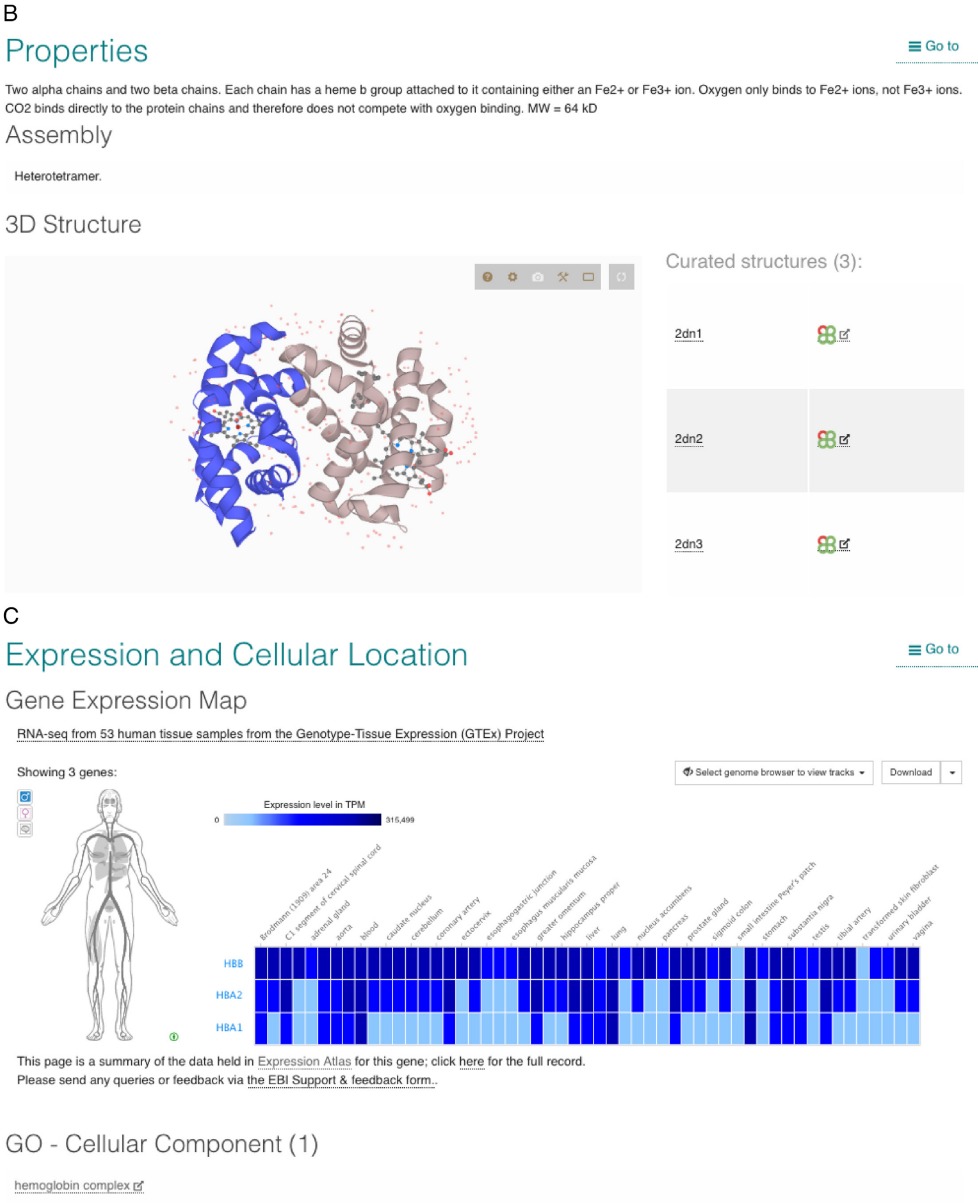
(**A**–**C**) Reactome, PDBe and Gene Expression Atlas tools as integrated in Complex Portal for Hemoglobin HbA complex.

Use of the component-based JS front-end technology enables the easy integration of external APIs, enabling Complex Portal entries to be enriched with data from collaborating resources (Figure 2). The Reactome viewer (14) allows browsing through pathways related to the complex of interest. Clicking on any element changes the focus of the diagram to this element and clicking on the Reactome symbol opens it in the Reactome website providing their full functionality. PDBe's LiteMol viewer (15) allows detailed investigation of 3D structures where these are available. Users can further investigate the original structural data entry via the link to the PDBe website. Finally, the Body Map tool from the Gene Expression Atlas displays tissue-specific gene expression data for all members of the complex for most multi-cellular species (16).

## DOWNLOAD OPTIONS

All complexes are available for download in the molecular interaction standard formats PSI-MI XML2.5 (17) and PSI-MI XML3.0 (18). PSI-MI XML2.5 is the older, more widely adopted format, but has limitations in the representation of complexes. Its schema was designed to represent experimental interactions, therefore an experiment description is required for each interaction. This is not appropriate for abstracted interactions such as curated complexes, where data from multiple publications may be combined, therefore the Complex Portal became an early adopter of the updated PSI-MI XML3.0 format, as being more appropriate for this data type.

Many visual applications now preferentially use JSON as an input format including the ComplexViewer which inputs newly-developed MI-JSON (13). This is written by the Complex Portal webservice (1) and *export* and *complex* methods have been added to the existing *search* and *details* methods (https://www.ebi.ac.uk/intact/complex-ws/).

Additionally, on request from our users, a tab-delimited format, ComplexTab, has been developed, modelled on the HUPO-PSI MITAB format (17). ComplexTab exports data in 18 columns (Table 1) listing details for each complex in a single, tab-delimited line with pipes separating elements in the same column. Fields including names, lists of synonyms, participants and cross-references, description, properties and assembly. It does not include any participant feature descriptions including binding regions or required PTMs as they cannot be simply assigned to the right complex member. If more detailed data is required the user is advised to use the MI-JSON or PSI-MI XML3.0 format.

**Table 1.** Descriptions for each of the 18 fields in the new ComplexTab format

Field name	Field description
**Unique identifier for complex**	The unique primary identifier of the complex in the Complex Portal database.
**Recommended name**	The most commonly used description for the complex.
**Aliases for complex**	All regularly used synonyms. Multiple annotations are separated by ‘|’.
**Taxonomy identifier**	NCBI taxonomy identifier for complex.
**Identifiers (and stoichiometry) of molecules in complex**	The list of molecules and their stoichiometry (in parenthesis) that make up the complex. For proteins, the accession should be UniProtKB, for chemical entities, ChEBI, and for RNA, RNAcentral. When the stoichiometry of a molecule is unknown, it is represented by (0). Multiple identifiers are separated by ‘|’.
**Confidence**	Evidence and Conclusion Ontology AC and term name.
**Experimental evidence**	Identifier of the experimental evidence for this complex in an IMEx member molecular interaction database, PDB or EMDB.
**GO annotations**	List of Gene Ontology terms the complex is annotated to. Multiple annotations are separated by ‘|’.
**Cross references**	List of accession codes for the complex in related databases, such as PDB, Reactome or MatrixDB, as well as links to diseases caused by changes in the complex. Multiple annotations are separated by ‘|’.
**Description**	A free-text description of the function(s) and role(s) of the complex.
**Complex properties**	A free-text description of the physical properties of the complex such as assembly and internal topology details, size, molecular weight or binding to co-factors.
**Complex assembly**	Description of the assembly type, such as Homodimer, or Heterohexamer.
**Ligand**	List of natural ligands of the complex. Multiple annotations are separated by ‘|’.
**Disease**	List of description of diseases caused by changes in the complex. Multiple annotations are separated by ‘|’.
**Agonist**	List of natural agonists of the complex. Multiple annotations are separated by ‘|’.
**Antagonist**	List of natural antagonists of the complex. Multiple annotations are separated by ‘|’.
**Comment**	List of comments that do not fit any of the other defined fields. Multiple annotations are separated by ‘|’.
**Source**	Group the original curator of the complex is associated with.

Access to the data is available in all formats described above via the <Programmatic Access> tile on the homepage and access to the PSI-MI XML and MI-JSON files from the header of each individual complex page. On the ftp site (ftp.ebi.ac.uk/pub/databases/intact/complex/current/), each complex is stored in a unique XML file, grouped into folders by species. ComplexTab data is available in a single file per species. The webservice provides a MI-JSON file per complex on demand. Download options for Search Results and the Basket are under development.

## COLLABORATION AND COMMUNITY INVOLVEMENT

The Complex Portal entries are designed to integrate data from multiple resources which hold information on these biologically important entities. Cross-references to UniProt provide information on the component proteins, whilst reciprocal cross-references in UniProt inform users as to the complex membership of individual proteins. Links to Reactome provide information on the role protein complexes play in pathways and cellular processes. All Reactome reactions have now been integrated into WikiData and use the Complex Portal ACs as primary complex identifiers where available (see https://www.wikidata.org/wiki/Q50250937). Links to ChEMBL enable the researcher to access a description of drugs which bind to and modulate the activity of a protein complex. The Complex Portal is a member of the Gene Ontology (GO) Consortium: Every complex is annotated to the relevant Gene Ontology terms for the complex as a whole, enabling hierarchical searching of the data set. The GO annotation guidelines have been updated to allow use of Complex Portal identifiers as GO annotation objects in GO editorial tools, such as Protein2GO. Annotations to Complex Portal identifiers are exported to and available through GO annotation files (GPAD, GPI) and can be searched and viewed via the web application QuickGO (https://www.ebi.ac.uk/QuickGO/search/CPX-591). The InterMine open source data warehouse system (19) has developed a pipeline to import protein complexes into the InterMine environment using the recently published JAMI library (20) to read and translate the data into interaction objects and export a MI-JSON file to the ComplexViewer. YeastMine and HumanMine are already making relevant data sets publicly available. The IMEx Consortium of interaction databases (21) now describe molecules that interact with complexes using Complex Portal entries as annotation objects.

As described above, the prioritized curation of complexes with a known complete, or partial, 3D structure has been undertaken as a collaboration with students from CEITEC (Masaryk University) and PDBe, and the yeast complexsome has been assembled in partnership with the Saccharomyces Genome Database and curators from the UniProt Consortium. UniProt curators have also contributed to the annotation of nematode complexes. We are interested in collaborating with other interest groups to continue to expand and improve this key reference resource and encourage potential collaborators to contact us via the yellow <Request> tile on the homepage or directly by emailing us at complexportal@ebi.ac.uk.

## RELATED RESOURCES

The Complex Portal focuses on stable, functional biological units which are the molecular machineries that drive biological processes. Compared to other macromolecular complex repositories, the Complex Portal uniquely adds details of nucleic acid and small molecule components of protein complexes, and describes the roles each contributes to complex function. This level of detail requires the collation of information abstracted from multiple publications. Evidence for the component content of a complex is linked, when available, to the detailed experimental curation of molecular interactions in the IMEx Consortium databases (21), a consortium of resources which systematically capture experimental interaction data from peer-reviewed journals. A complex may be described within a single interaction in an IMEx record, or may need an expert biocurator to assemble evidence from multiple records.

The CORUM database of mammalian complexes (22) defines complexes based on experimental evidences found in a single publication, akin to classic protein-protein interaction databases. Its coverage for mammalian complexes is currently larger than Complex Portal while Complex Portal provides a broader species range. The overlap of complexes between the 2 resources cannot be directly compared as their definition of complexes is not the same: several experimentally-evidenced complexes from CORUM may be combined into one Complex Portal entry while Complex Portal provides separate entries for variants (see Section “Variants, molecule sets and complexes as participants” above).

## SUMMARY AND FUTURE PLANS

The Complex Portal has been designed with 3 major use cases in mind. Firstly, the description of individual protein complexes to allow information on these biological entities to be integrated across multiple biomedical resources. We will continue to add cross-references and further improve our API in order to enable this. Secondly, the completion of the entire set of known protein complexes for key model organisms will enable these to be identified in large-scale data sets. ComplexTAB has been designed as an easy-to-parse option to provide data for this purpose. *Saccharomyces cerevisiae* is the first organism for which we believe we have achieved a complete representation of current knowledge but we anticipate more complexes will be identified in this organism as experimental techniques become more sensitive and we will continue to enhance this set as information becomes available. We look to complete the first bacterial complexsome, for *Escherichia coli*, in 2019 and will continue to work towards a fuller representation of the human and murine complex sets. Thirdly, we look to contribute to a broader understanding of complex evolution and development across a range of taxonomic species by identifying how complex content and function alter with the complexity of the host organism. We are currently developing a mechanism by which orthologous and paralogous complexes can be identified and a tool to subsequently visualize these relationships and will be making this publicly available on the Complex Portal website in the near future.

We constantly try to improve our databases and services in terms of accuracy and representation and actively encourage user feedback. If you have any questions or suggestions please contact the Molecular Interaction Team via the yellow <Request> tile on the homepage or email us directly at complexportal@ebi.ac.uk. Information about curation is provided at https://www.ebi.ac.uk/complexportal/documentation. Extensive training material on how to best use our resource is available at https://www.ebi.ac.uk/training?ebi-topic%5Bsystems%5D=systems.

### Computational Resources

The Complex Portal is a community project. Developers can contribute to the code at https://github.com/Complex-Portal/complex-portal-view.

Data can be accessed either via our ftp site (ftp.ebi.ac.uk/pub/databases/intact/complex/current/) or our REST API (https://www.ebi.ac.uk/intact/complex-ws/).
